# Specificity of peptidases secreted by filamentous fungi

**DOI:** 10.1080/21655979.2017.1373531

**Published:** 2017-09-21

**Authors:** Youssef Ali Abou Hamin Neto, Nathália Gonsales da Rosa Garzon, Rafael Pedezzi, Hamilton Cabral

**Affiliations:** School of Pharmaceutical Sciences of Ribeirão Preto, University of São Paulo, Ribeirão Preto, Brazil

**Keywords:** catalytic site, cooperativity subsite, filamentous fungi, peptidase, specificity

## Abstract

Peptidases are enzymes that cleave peptide bonds, yielding proteins and peptides. Enzymes in this class also perform several other functions, regulating the activation or inactivation of target substrates via proteolysis. Owing to these functions, peptidases have been extensively used in industrial and biotechnological applications. Given their potential functions, it is important to optimize the use of these enzymes, which requires determination of the specificity of each peptidase. The peptidase specificity must be taken into account in choosing a peptidase to catalyze the available protein source within the desired application. The specificity of a peptidase defines the profile of enzyme–substrate interactions, and for this the catalytic site and the arrangement of the amino acid residues involved in peptide bond cleavage need to be known. The catalytic sites of peptidases may be composed of several subsites that interact with amino acid residues for proteolysis. Filamentous fungi produce peptidases with varying specificity, and here we provide a review of those reported to date and their potential applications.

All biological organisms are supported by a continuing series of chemical reactions, and thus by enzymes that catalyze these reactions.^1^ For many centuries, people have used these enzymes in many processes, for production of food and other materials. Currently, these biomolecules play an important role in various industrial applications, owing to characteristics such as efficiency, rapidity, the ability to operate at low substrate concentrations, low toxicity, high specificity, and the ease of interrupting the reaction.^2^ In addition, biocatalysts are renewable; they thus provide many advantages compared to chemical catalysts.^3^

Peptidases are enzymes that are also known as proteases, proteinases, and proteolytic enzymes (International Union of Biochemistry & Molecular Biology).^4^ These macromolecules catalyze the cleavage of peptide bonds in polypeptide chains in the presence of water, and are therefore classified as hydrolases.^5^

In general, these enzymes catalyze reactions that are important to various physiological processes, such as the cell cycle, cell growth and differentiation, apoptosis, and other functions.^6^

Microorganisms are the main source of peptidases used in large-scale production, due to their rapid growth and the current good understanding of the organismal machinery, characteristics that facilitate the production of new recombinant enzymes.^7-8^

Peptidases isolated from microorganisms can act at a wide range of pH and temperature values, and can catalyze many kinds of substrates; they have thus become an important tool in industrial applications.^5^ Moreover, they have been exploited in various industrial sectors, such as: peptide synthesis, food and feed production, leather and textile processing, medical and pharmaceutical production, and others.^9^

Industrial applications are related to biochemical characteristics and specificity, which depends on the amino acid residues present at the catalytic site. Peptidases can be classified by catalytic mechanism as serine, cysteine, aspartyl, threonine, glutamic or metallopeptidases.^10^ The catalytic site of an enzyme performs two functions: substrate binding and catalysis. Thus the efficiency of these functions determines the activity of an enzyme in relation to a particular substrate, and it also determines enzyme specificity.^11^

According to Schechter and Berger (1967), the catalytic sites of peptidases can be subdivided into subsites, which each accommodate a corresponding amino acid residue of the substrate.^11^ These subsites are numbered according to the amino acid residue of the polypeptide chain with which they interact, and the substrate is named according to the cleaved site (P_1_-P′_1_), S_n_-S_3_-S_2_-S_1_-S′_1_-S′_2_-S′_3_ –S′_n_ and P_n_-P_3_-P_2_-P_1_-P′_1_-P′_2_-P′_3_-P′_n_, respectively (Fig. 1).

**Figure 1.**
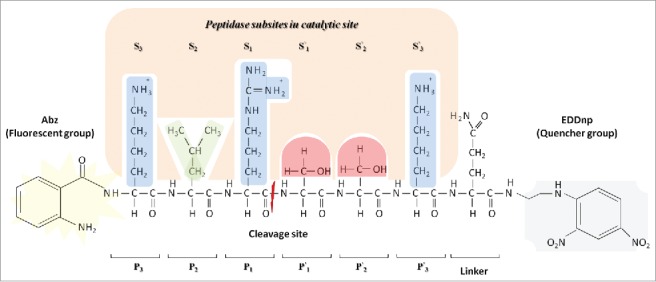
Model proposed by Schechter and Berger (1967)^11^ which shows the peptidase subsites in a catalytic site. The catalytic site of the peptidase is divided into subsites, named S_n,_ S_3_, S_2_, S_1_, S′_1_, S′_2_, S′_3_, and S′_n_ based on cleavage point in the polypeptide chain, the amino acid residues are named P_n,_ P_3_, P_2_, P_1_, P′_1_, P′_2_, P′_3_ and P′_n_ based on the corresponding subsites. Subsite mapping determines the peptidase specificity. This illustration represents the interaction of the substrate Abz-KLRSSKQ-EDDnp with peptidase subsites. Side chain properties are shown by color: Blue indicates a positive polar side chain from P_3_, P_1_ and P′_3_ amino acids residues; green indicates a hydrophobic side chain from P_2_ amino acid residue; red indicates a polar neutral side chain from P′_1_ and P′_2_ amino acids residues. Quencher group EDDnp: ethylene diamine 2,4-dinitrophenyl. Fluorescent group Abz: O-aminobenzoyl.

In addition, the subunits of the catalytic site vary in their ability to bind amino acid residues; some are restricted to one or a few amino acids with similar chemical characteristics, whereas others can interact with all of them. Thus, in a simplified representation of a peptidase catalytic site, there are fixed subsite numbers with specific preferences for different amino acids, allowing peptidases to discriminate between cleavage sites, generating specificity.^6^

The ability of this peptidase to interact with a specific polypeptide chain sequence is faithful to original substrate structure of biological processes, which the enzyme belongs.^12^ Considering that peptidase specificity is determined by substrate recognition mediated by amino acid sequence at the catalytic site, subsite mapping by peptide libraries is essential for characterization to allow deduction of enzyme preference by substrate. In addition to specificity, which is determined by the catalytic site, some substrates, as native proteins, present structural restrictions, making coupling and cleavage more difficult.^13^

Among the methods used to map subsites, some use substrates containing a fluorophore group, such as the 7–methoxycoumarin–4–acetic acid (MCA) probe. This is a rapid methodology for specificity screening, but only reveals unprimed-side (S_1_, S_2_, S_3_ and S_n_) specificity. Alternately, libraries of fluorescence resonance energy transfer (FRET) substrates with known amino acid sequences and a donor (Abz) and a quencher (EDDnp) group (Fig. 1) are available, which allow determination of the specificity of both the unprimed (S_n_, S_3_, S_2_, and S_1_) and primed sides (S′_1_, S′_2_, S′_3_ and S′_n_), increasing precision.^12^^,^^14^

Specificity assays yield important information about enzyme function and can suggest a possible structure of a catalytic site, which contributes to select the optimal substrate and to designing highly selective biological probes.^12^^,^^15^

In addition to interactions between each subsite and its corresponding amino acid residue, neighboring substrate amino acids can exert a negative or positive influence on the enzyme subsite. This subsite cooperativity has been observed in several studies of peptidase specificity, but has been neglected in study of the interaction between catalytic site and polypeptide chain sequence. Cooperativity has been reported between nearby subsites, such as the S_1_, S_2_, and S_3_ subsites of thrombin, as well as between distant subsites, such as S_1_ and S_4_ of subtilisin.^6^^,^^16-17^

Cooperativity is critical for complete elucidation of the relationships between catalytic sites and their effect on the specificity of proteolysis. Cooperation between one substrate and another at the molecular level is important to understand how the catalytic site recognizes and selects a substrate for cleavage. In the future, studies using combined kinetic and structural approaches will provide a much greater understanding of the mechanisms underlying subsite cooperativity.^6^

Researchers have used various kinds of substrates to evaluate the kinetic parameters and consequently the catalytic efficiency and specificity of various peptidases (Table 1).

**Table 1.** List of peptidases from filamentous fungi and their specificities based on primed and unprimed catalytic subsites according to different substrates.

		Unprimed Subsites					Primed Subsites						
Microorganism	Classification	**S_5_**	**S_4_**	**S_3_**	**S_2_**	**S_1_**	**S^’^_1_**	**S^’^_2_**	**S^’^_3_**	**S^’^_4_**	**S^’^_5_**	Substrate	Ref.
*Fusarium equiseti*	Subtilase-like	ND	Asn	Gly	Thr	His	Tyr	Gly	Lys	Gly	ND	Hydrolysis profile of the β-casein, cytochrome c and ubiquitin	[18]
*Aspergillus fumigatus*	Sedolisin (SedA)	ND	ND	ND	ND	ND	ND	ND	ND	ND	ND	Resorufin-labeled casein	[19]
*Talaromyces emersonii*	Prolyl aminopeptidases	ND	ND	ND	ND	Pro	ND	ND	ND	ND	ND	Chromogenic substrates (Pro-*p*NA; Ala- *p*NA; Val- *p*NA; Leu- *p*NA; Phe- *p*NA; Gly- *p*NA or Gly-Pro- *p*NA)	[20]
*Aspergillus oryzae*	Dipeptidyl peptidases DppB	ND	ND	ND	Ala	Pro	ND	ND	ND	ND	ND	Chromogenic substrates (Arg-Pro-*p*NA; Ala-Pro-*p*NA and Gly-Pro-*p*NA)	[21]
*Aspergillus oryzae*	Dipeptidyl peptidases DppE	ND	ND	ND	Gly	Phe	ND	ND	ND	ND	ND	Chromogenic substrates (Lys-Ala-*p*NA; Ala-Ala-*p*NA and Gly-Phe-*p*NA)	[21]
*Aspergillus oryzae*	Dipeptidyl peptidases DppF	ND	ND	ND	Gly	Phe	ND	ND	ND	ND	ND	Chromogenic substrates (Lys-Ala-*p*NA; Ala-Ala-*p*NA and Gly-Phe-*p*NA)	[21]
*Aspergillus fumigatus*	Sedolisin (SedB)	ND	ND	Phe Ala	Pro Ala	Ala Phe	ND	ND	ND	ND	ND	Chromogenic substrate Ala-*p*NA, Gly-Pro-*p*NA, Ala-Ala-*p*NA, Phe-Pro-Ala-*p*NA, Ala-Ala-Pro-*p*NA, Ala-Ala-Phe-*p*NA or Ala-Ala-Pro-Leu-*p*NA	[19]
*Aspergillus fumigatus*	Sedolisin (SedB)	ND	ND	Ala Asp	Pro Arg	Gly Ile	Asp Tyr	Arg Val	Ile His	Tyr Pro	Val Phe	Synthetic substrate Ala-Pro-Gly-Asp-Arg-Ile-Tyr-Val-His-Pro-Phe	[19]
*Aspergillus fumigatus*	Sedolisin (SedC)	ND	ND	Phe Ala	Pro Ala	Ala Phe	ND	ND	ND	ND	ND	Chromogenic substrate Ala-*p*NA, Gly-Pro-*p*NA, Ala-Ala-*p*NA, Phe-Pro-Ala-*p*NA, Ala-Ala-Pro-*p*NA, Ala-Ala-Phe-*p*NA or Ala-Ala-Pro-Leu-*p*NA	[19]
*Aspergillus fumigatus*	Sedolisin (SedD)	ND	ND	Phe Ala	Pro Ala	Ala Phe	ND	ND	ND	ND	ND	Chromogenic substrate Ala-*p*NA, Gly-Pro-*p*NA, Ala-Ala-*p*NA, Phe-Pro-Ala-*p*NA, Ala-Ala-Pro-*p*NA, Ala-Ala-Phe-*p*NA or Ala-Ala-Pro-Leu-*p*NA	[19]
*Talaromyces emersonii*	Glutamic peptidase	ND	ND	ND	ND	ND	ND	ND	ND	ND	ND	Hydrolysis profile of 16 substrates analyzed by MALDI-TOF MS	[22]
*Rhizomucor miehei*	Aspartic peptidase	ND	ND	Lys	Asn	Arg	Met	Lys	Met	ND	ND	FRET peptides	[23]
*Rhizomucor miehei*	Aspartic peptidase	ND	ND	Lys	Ser	Phe	Met	Ala	Ile	ND	ND	FRET peptides with κ-casein sequence	[23]
*Phanerochaete chrysosporium*	Aspartic peptidase	ND	ND	Lys	Leu	Arg	Ser	Ser	Lys	ND	ND	FRET peptides	[24]
*Aspergillus terreus*	Serine peptidase	ND	ND	Ser	Ile	Tyr	Ser*	Ser*	Lys*	ND	ND	FRET peptides	[25]
*Myceliophthora thermophila*	Serine peptidase	ND	ND	Met	Val	Thr	Ala	Ala	Ser	ND	ND	FRET peptides	[26]
*Penicillium waksmani*	Serine peptidase	ND	ND	Leu	Ile	Asp	Hys	Phe	Arg	ND	ND	FRET peptides	[27]
*Myceliphthora sp*	Serine peptidase	ND	ND	Lys	Phe	Ile	Ser	Phe	Lys	ND	ND	FRET peptides	[28]
*Eupenicillium javanicum*	Metallopeptidase	ND	ND	Val	Val	Arg	Tyr	Ile	Tyr	ND	ND	FRET peptides	[29]
*Thermoascus aurantiacus*	Metallopeptidase	ND	ND	Lys*	Leu*	Arg	Ser*	Ser*	Lys*	ND	ND	FRET peptides	[30]
*Phanerochaete chrysosporium*	Cysteine peptidase	Arg	Gln	Phe	Arg	Lys	Lys	ND	ND	ND	ND	FRET peptides	[31]

*Fixed amino acid; ND: not determined; FRET: fluorescence resonance energy transfer. Ref.: Reference.

Peptidases can be divided into various classes, and each one has a specificity to a determined substrate. Juntunem and collaborators (2015) described recombinant production in *Trichoderma reesei* of a new extracellular subtilisin-like enzyme from *Fusarium equiseti*. They determined some of the biochemical properties and substrate specificity (β-casein, cytochrome c, and ubiquitin) of this purified peptidase. The substrate β-casein was fully digested after 5 min of incubation at pH 6.8 and 9.0; in contrast, cytochrome c and ubiquitin were refractory to peptidase-mediated catalysis under the same conditions. The peptides generated from cleavage of the substrates, using different pH values and incubation times, were subjected to mass spectrometry analysis. The normalized data were applied in a matrix which was used to determine substrate specificity. For unprimed subsites, the best hydrolysis profile was observed for His (polar positive), Thr (hydrophobic), Gly (hydrophobic), and Asn (polar neutral) at the P_1_, P_2_, P_3_, and P_4_ positions, respectively. For primed subsites, the best hydrolysis profile was observed for Tyr (hydrophobic), Gly (hydrophobic), Lys (polar positive), and Gly (hydrophobic) at the P′_1_, P′_2_, P′_3_, and P′_4_ positions, respectively. The cleavage patterns indicated a preference for amino acid residues with non-charged side chains. Overall, this peptidase showed a broad substrate specificity and only some restrictions for each subsite. Moreover, this recombinant enzyme showed good performance in detergent applications for stain removal. Together, these properties highlight the industrial potential of this subtilisin-like peptidase.^18^

*Aspergillus fumigatus* produces four different sedolisins (SedA, SedB, SedC, and SedD), peptidases that act as endo- (SedA) or exopeptidases (SedB, SedC, and SedD). These enzymes were heterologously expressed in *Pichia pastoris* and characterized by Reichard and collaborators (2006). SedA was characterized as an endopeptidase, capable of hydrolyzing casein, but showed no activity against mono-, di-, tri-, or tetrapeptide substrates. SedB, SedC, and SedD showed activity against Phe-Pro-Ala-pNA or Ala-AlaPhe-pNA substrates (hydrophobic), and were thus classified as tripeptidil peptidases. SedB was chosen for protein purification and was assayed with an Ala-Pro-Gly-Asp-Arg-Ile-Tyr-Val-His-Pro-Phe substrate. Mass spectrometry analysis revealed the liberation of Ala-Pro-Gly, Asp-Arg-Ile, and Tyr-Val-His-Pro-Phe peptides. Although it showed a preference for Gly or Ile (hydrophobic with aliphatic chain side) at the P_1_ position, SedB bypassed proline (hydrophobic with a cyclic chain side) when it was at the P_1_ or P′_1_ position, and did not hydrolyze the peptide. This was also observed with Tyr-Val-His-**Pro**-Phe and Ala-Ala-**Pro**-pNA peptides, which were not hydrolyzed.^19^

Mahon and collaborators (2009) studied a prolyl aminopeptidase from *Talaromyces emersonii*, which is intracellularly produced. The enzyme was purified by fractionation and five chromatographic steps. They tested different substrates, but the maximal activity was observed with Pro-*p*NA at P_1_ position.^20^

Maeda and collaborators (2016) performed recombinant production of the dipeptidyl peptidases DppB, DppE, and DppF, and determined their kinetic parameters by modifications of the amino acid residues at the P_2_-P_1_ substrate positions. These dipeptidyl peptidases demonstrated differing activities for the various substrates tested. DppB, DppE, and DppF from *Aspergillus oryzae* provided the highest rate of hydrolysis for Arg-Pro-pNA, Gly-Phe-pNA, and Lys-Ala-pNA substrates, respectively. Moreover, the kinetic parameters were used to define the catalytic efficiency. Comparative analysis of DppB using Pro at the P_1_ position demonstrated that the activity was modulated by the S_2_ subsite and that charged and bulky amino acid residues, such as alanine, were preferred at the P_2_ position. DppE and DppF showed similar profiles, with a preference for the apolar amino acid residue Phe at the P_1_ position; amino acid residues at the P_2_ position had little effect.^21^

O'Donoghue and collaborators (2008) characterized a glutamic peptidase TGP1 from Talaromyces emersonii. The enzyme showed only endopeptidase activity, once it was capable of hydrolysis, with histidine and leucine at positions P1 and P′1, respectively, and only when P′1 was not the last residue. The results suggest that substrate recognition is important from S3 to S′3. They tested 16 peptides and identified 32 cleavage points by MALDI-TOF MS analysis. TGP1 has a wide spectrum of substrate recognition, but a pattern was observed: the residues at the P1 position are larger than those at the P′1 position.^22^

Using modification of the amino acid residues at the P_3_-P_2_-P_1_-P′_1_-P′_2_-P′_3_ FRET substrate positions, the kinetic parameters of a rhizopuspepsin-like aspartic peptidase from *Rhizomucor miehei* were recently identified. Primed S′_1_, S′_2_, and S′_3_ subsites demonstrated greatest catalytic efficiency for non-polar, basic, and non-polar amino acid residues, respectively, and also for aromatic amino acid residues. The catalytic efficiency of the unprimed S_1_, S_2_, and S_3_ subsites was differentially modulated, and demonstrated a preference for basic, neutral polar, and basic amino acids residues, respectively. The replacement of Lys for Leu at P_3_ position (Abz-**L**LRSSKQ-EDDnp) provided the best catalytic efficiency, as the hydrophobic Lys provided a better interaction between the catalytic site and peptide than the polar Leu, with a k_cat_/K_M_ = 1294 mM^−1^·s^−1^, with a cleavage site between P_1_↓ P′_1_. In general, primed subsites presented lower selectivities than unprimed subsites. Additionally, rhizopuspepsin-like from *R. miehei* demonstrated to cleave substrates containing a *k*-casein sequence (Abz-LSFMAIQ-EDDnp), with a coagulant activity higher than its proteolytic activity. Given these potential activities, the authors proposed that this aspartyl peptidase can be used for peptide synthesis or casein hydrolysis, especially in cheese production.^23^

Silva et al. (2017) studied *Phanerochaete chrysosporium,* a well-known producer of lignocellulolytic enzymes, using an innovative approach to characterize the kinetic parameters of an aspartic peptidase from *P. chrysosporium*. Primed S′_1_, S′_2_, and S′_3_ subsites demonstrated better catalytic efficiency for neutral polar, neutral polar, and basic amino acid residues, respectively. On the other hand, unprimed S_1_, S_2_, and S_3_ subsites showed a preference for basic, non-polar, and basic amino acid residues, respectively. The authors proposed that the S_1_ subsite showed a preference for amino acid residues with basic and non-cyclic side chains. The higher catalytic efficiency was 4792 mM^−1^·s^−1^ without replacement (Abz-KLRSSKQ-EDDnp) at the substrate, and the cleavage site was between P_1_↓ P^′^_1_.^24^

Biaggio et al. (2016) studied the specificity of the unprimed subsites of a serine peptidase produced by *Aspergillus terreus*. Using FRET synthetic peptides with replacements at the P_1_, P_2_, and P_3_ positions, the authors determined the S_1_, S_2_, and S_3_ subsite catalytic efficiency, which was also used as parameter to determine the subsite selectivity. The S_1_ subsite had a preference for aromatic amino acid residues, the best catalytic efficiency (363.4 mM^−1^·s^−1^) was obtained with Tyr at the P_1_ position (Abz-KLYSSKQ-EDDnp), and the cleavage site was between P_1_↓ P′_1_, although the presence of Ile at the P_1_ position prevented hydrolysis. The S_2_ subsite showed low hydrolysis of all substrates and the best catalytic efficiency (98 mM^−1^·s^−1^) was obtained with the non-polar amino acid residue Ile at the P_2_ position (Abz-K**I**RSSKQ-EDDnp). Furthermore, all substrates with changes at the P_3_ position were hydrolyzed and the polar amino acid residue Ser (Abz-**S**LRSSKQ-EDDnp) at P_3_ position showed the best catalytic efficiency (318.7 mM^−1^·s^−1^). Due to their hydrolysis profiles, the selectivity of the S_1_ and S_3_ subsites was less than that of the S_2_ subsite.^25^

The fungus *Myceliophthora thermophila* produces a serine peptidase under submerged bioprocessing, and the kinetic parameters of the enzyme were evaluated with FRET substrates, with replacement of the amino acids at the P_1,_P_2,_P_3,_P′_1,_P′_2_, and P′_3_ positions of the substrate. In general, both sides (unprimed and primed) accepted hydrophobic amino acids. The authors observed the replacement of serine (hydrophobic) for alanine (hydrophobic with a smaller side chain) at the P′_2_ position (Abz-KLRS**A**KQ-EDDnp); catalytic efficiency was higher, with 18200 mM^−1^·s^−1^ and the cleavage site was between P_1_↓ P′_1_.^26^

Graminho and collaborators (2013) purified a serine peptidase from submerged bioprocessing of *Penicillium waksmanii*. The enzyme parameters were evaluated with FRET substrates with replacement of amino acids at P_1,_P_2,_P_3,_P′_1,_P′_2_, and P′_3._ In general, both sides (unprimed and primed) accepted non-polar amino acids. The results showed the replacement of leucine (hydrophobic) for isoleucine (hydrophobic with a larger side chain) at the P_2_ position of the substrate (Abz-K**I**RSSKQ-EDDnp) resulted in a better interaction with its corresponding catalytic subsite, and consequently, a higher catalytic efficiency, of 10666 mM^−1^·s^−1,^ and the cleavage site was between P_1_↓ P′_1_.^27^

Zanphorlin and collaborators (2011) studied a peptidase secreted by *Myceliophthora* genus. The fungus *Myceliophthora* sp. produces a serine peptidase under solid state bioprocessing. Using FRET peptides, the kinetic parameters showed differences in catalytic efficiency following replacement of amino acids at the P_1,_P_2,_P_3,_P′_1,_P′_2_, and P′_3_ positions of the substrate. The presence at the P_1_ position (Abz-KL**X**SSKQ-EDDnp) of Ile, Met, or Trp yielded a higher catalytic efficiency than Arg (Abz-KL**R**SSKQ-EDDnp), with values of 1275, 676, and 639 mM^−1^·s^−1^, respectively. The P_1_ position showed a preference for hydrophobic amino acids. The presence of Phe and Pro, rather than Leu, at the P_2_ position (Abz-K**L**RSSKQ-EDDnp) promoted a high level of catalytic efficiency, at 683, 299, and 133 mM^−1^·s^−1^, respectively. These amino acids are hydrophobic but only Leu has an aliphatic lateral chain, which may influence accommodation at the catalytic site. The exchange of Ser for Phe, His, or Leu at P′_2_ improved catalytic efficiency, with rates of 133, 332, 261, and 164 mM^−1^·s^−1^. The replacement of the original amino acids at P_3_, P′_1_, and P′_3_ decreased catalytic efficiency. The authors suggest that all substrates were found to be cleaved between P_1_ and P′_1_.^28^

According to Hamin Neto et al. (2016), the fungus *Eupenicillium javanicum* produces a metallopeptidase by solid state bioprocessing, and the authors evaluated the replacement of amino acids at P_1,_P_2,_P_3,_P′_1,_P′_2_ and P′_3_ positions of the substrate. In general, the unprimed side accepted hydrophobic amino acids, and the primed side had a preference for tyrosine. The replacement of serine (hydrophobic) for tyrosine (polar non-charged) at the P′_1_ position (Abz-KLR**Y**SKQ-EDDnp) and its interaction with S′_1_at the catalytic site provided higher catalytic efficiency for metallopeptidase, with k_cat_/K_M_ = 87849 mM^−1^·s^−1^. The substrate cleavage site was also evaluated; the FRET peptide was cleaved between P′_1_↓ P′_2_.^29^

Merheb-Dini and collaborators (2009) studied the kinetic parameters of a metallopeptidase secreted by the fungus *Thermoascus aurantiacus* in solid state bioprocessing, and used FRET substrates for replacement of the amino acids at P_1_. The researchers observed the highest catalytic efficiency with Arg (Abz-KL**R**SSKQ-EDDnp) followed by Lys, at 30.1 and 14.9 mM^−1^·s^−1^, respectively. Both amino acids have polar basic properties, but Arg has longer side chain than Lys.^30^

*Phanerochaete chrysosporium* produces a lysine-dependent cysteine peptidase, under submerged bioprocessing FRET substrates were used to evaluate the kinetic parameters and cleavage site of this enzyme. The substrate Abz-**XXXXX**KQ-EDDnp was cleaved between X↓K, where X is any amino acid, with the exception of the substrate Abz-KLRS↓K↓KQ-EDDnp. Analysis of each subsite showed that when P′_1_ is not lysine, the reaction does not occur. The efficiency of catalysis observed with replacement of amino acid residues at P_1_ showed a preference for basic amino acids (Lys, 3500 mM^−1^·s^−1^ and Arg, 3222 mM^−1^·s^−1^) rather than polar uncharged (Ser, 1650 mM^−1^·s^−1^). Substitutions at P_2_ with basic amino acids (Arg and Tyr) produced higher catalytic efficiency than polar uncharged (Ser), 3889, 3428.5 and 1650 mM^−1^·s^−1^, respectively. Amino acid substitution at P_3_ showed high catalytic efficiency with Phe (hydrophobic with an aromatic ring) rather than Arg. At the P_4_ position the replacement of Leu (hydrophobic) for Gln (polar uncharged) and Arg (basic) increased the catalytic efficiency from 1650 to 2250 and 2000 mM^−1^·s^−1,^ respectively. In addition, Arg rather than Lys at P_5_ produced a higher catalytic efficiency, with 1786 mM^−1^·s^−1^ and at P′_1_ the substrate was only cleaved when Lys was present. Other substrates such as fibrinogen, collagen, and k-casein clotting fragments were tested, but the reaction did not occur owing to the absence of lysine at the P′_1_ position. This peptidase showed poor catalytic efficiency for acidic residues D and E. This enzyme also showed clear cooperativity between subsites (induced fit) since the S′_1_ position is restrictive for lysine, but S_5_ increases the hydrolysis potential when the substrate residue is basic.^31^

The determination of specificity and subsite cooperativity of fungal peptidases can improve the biotechnological applications and the current trends approached the use of modern techniques for the better exploitation of knowledge. The use of computational design allows the remodeling of enzyme specificity to increase this activity.^32^ Moreover, the specific cleavage from peptidases can be maintained whereas their biochemical properties can be modified using recombinant DNA techniques, and to diversify their suitable processes.^33^

In food industry, the use of peptidases able to provide the Phe_105_-Met_106 _specific cleavage in κ-casein is essential for milk coagulation and cheese production.^33^ The peptidases specificity also enabled the modulation of the chemical and nutritional properties and provided a better defined composition. The protein hydrolysis can be used for improve the taste and solubility, moreover to generate peptides with biological activity,^34^ such as antihypertensive, antimicrobial, antioxidant, opioid and immunomodulatory.^35^

Pharmaceutical industries have been produced some peptidases to diseases therapy related to abnormalities or deficiencies of some enzymes and have been looked for new therapeutic targets both based on peptidase specificity.^36^

Proteomic is another field that specific peptidases have been used, trypsin is the peptidase used to prepare the sample for mass spectrometry, and this enzyme may impose certain limits in protein identification, therefore there is a search for new peptidases with high specificity to replace it.^37^

Filamentous fungi have been produced many products with biotechnological potential, among these products there are peptidases, these enzymes catalyze different polypeptides chain according to their properties. Some peptidases application are based on specific substrate cleavage, wide reactions conditions, inhibitors and activators.

Thus, the knowledge of biochemical characteristics, cleavage site, specificity and subsite cooperativity of fungal peptidases is important to determine the ideal substrate and reaction conditions, consequently, it allows to apply each peptidase in certain areas, as medical, pharmaceuticals, foods, detergents, leather, silk and chemical products.

## References

[1] Copeland AR A brief history of enzymology, In: Enzymes: A Practical Introduction to Structure, Mechanism, and Data Analysis. New york, A John Wiley & Sons, Inc 2000 P. 1-10.

[2] Sanchez S, Demain AL Enzymes and bioconversions of industrial, pharmaceutical, and biotechnological significance. Org Process Res Dev. 2011;15(1):224-230. doi:10.1021/op100302x

[3] Jemli S, Ayadi–Zouari D, Hlima HB, Bejar S Biocatalysts: application and engineering for industrial purposes. Crit Rev Biotechnol. 2016;36(2):246-58. doi:10.3109/07388551.2014.950550. PMID:25373789 25373789

[4] Barrett AJ Bioinformatics of proteases in the MEROPS database. Curr Opin Drug Discov Devel. 2004; 7(3):334-341. 15216937

[5] Srilakshmi J, Madhavi J, Lavanya S, Ammani K Commercial potential of fungal protease: Past, present and future prospects. J Pharm Biol Sci. 2015;2(4):218-234.

[6] Ng NM, Pike RN, Boyd SE Subsite cooperativity in protease specificity. Biol Cem 2009;390(5/6):401-407. 10.1515/BC.2009.065 19361286

[7] Rao MB, Tanksale AM, Ghatge MS, Deshpande VV Molecular and biotechnological aspects of microbial proteases. Microbiol Mol Biol Rev. 1998;62(3):597-635. PMID:9729602 9729602 10.1128/mmbr.62.3.597-635.1998 PMC98927

[8] Chanalia P, Gandhi D, Jodha D, Singh J Applications of microbial proteases in pharmaceutical industry. Rev Med Microbiol. 2011;22(4):96-101. doi:10.1097/MRM.0b013e3283494749.

[9] Gupta R, Beg QK, Lorenz P Bacterial alkaline proteases: molecular approaches and industrial applications. Appl Microbiol Biotechnol. 2002;59(1):15-32. doi:10.1007/s00253-002-0975-y. PMID:12073127 12073127

[10] Rawlings ND, Barrett AJ, Bateman A Asparagine peptide lyases: a seventh catalytic type of proteolytic enzymes. J Biol Chem. 2011;286(44):38321-38328. doi:10.1074/jbc.M111.260026. PMID:21832066 21832066 PMC3207474

[11] Schechter I, Berger A On the size of the active site in proteases. I Papain Biochem Biophys Res Commun. 1967;27(2):157-162. doi:10.1016/S0006-291X(67)80055-X. PMID:6035483 6035483

[12] Petrassi HM, Williams JA, Li J, Tumanut C, Ek J, Nakai T, Masick B, Backes BJ, Harris JL A strategy to profile prime and non–prime proteolytic substrate specificity. Bioorg Med Chem Lett. 2005;15(12):3162-3166. doi:10.1016/j.bmcl.2005.04.019. PMID:15878267 15878267

[13] auf dem Keller U, Schilling O Proteomic techniques and activity–based probes for the system–wide study of proteolysis. Biochimie. 2010;92(11):1705-1714. doi:10.1016/j.biochi.2010.04.027. PMID:20493233 20493233

[14] Korkmaz B, Attucci S, Juliano MA, Kalupov T, Jourdan ML, Juliano L, Gauthier F Measuring elastase, proteinase 3 and cathepsin G activities at the surface of human neutrophils with fluorescence resonance energy transfer substrates. Nat Protoc. 2008;3(6):991-1000. doi:10.1038/nprot.2008.63. PMID:18536646 18536646

[15] Diamond SL Methods for mapping protease specificity. Curr Opin Chem Biol. 2007;11(1):46-51. doi:10.1016/j.cbpa.2006.11.021. PMID:17157549 17157549

[16] Grøn H, Breddam K Interdependency of the binding subsites in subtilisin. Biochemistry. 1992;31(37):8967-71. doi:10.1021/bi00152a037. PMID:1390683 1390683

[17] Grøn H, Meldal M, Breddam K Extensive comparison of the substrate preferences of two subtilisins as determined with peptide substrates which are based on the principle of intramolecular quenching. Biochemistry. 1992;31(26):6011-8. doi:10.1021/bi00141a008. PMID:1627543 1627543

[18] Juntunen K, Mäkinen S, Isoniemi S, Valtakari L, Pelzer A, Jänis J, Paloheimo M A New Subtilase–Like Protease Deriving from *Fusarium equiseti* with High Potential for Industrial Applications. Appl Biochem Biotechnol. 2015;177(2):407-430. doi:10.1007/s12010-015-1752-6. PMID:26178876 26178876

[19] Reichard U, Léchenne B, Asif AR, Streit F, Grouzmann E, Jousson O, Monod M Sedolisins, a new class of secreted proteases from *Aspergillus fumigatus* with endoprotease or tripeptidyl–peptidase activity at acidic pHs. Appl Environ Microbiol. 2006;72(3):1739-48. doi:10.1128/AEM.72.3.1739-1748.2006. PMID:16517617 16517617 PMC1393174

[20] Mahon CS, O'Donoghue AJ, Goetz DH, Murray PG, Craik CS, Tuohy MG Characterization of a multimeric, eukaryotic prolyl aminopeptidase: an inducible and highly specific intracellular peptidase from the non–pathogenic fungus *Talaromyces emersonii*. Microbiology. 2009;155(Pt 11):3673-82. doi:10.1099/mic.0.030940-0. PMID:19556294 19556294 PMC2888130

[21] Maeda H, Sakai D, Kobayashi T, Morita H, Okamoto A, Takeuchi M, Kusumoto K, Amano H, Ishida H, Yamagata Y Three extracellular dipeptidyl peptidases found in *Aspergillus oryzae* show varying substrate specificities. Appl Microbiol Biotechnol. 2016;100(11):4947-4958. doi:10.1007/s00253-016-7339-5. PMID:26846741 26846741

[22] ODonoghue AJ, Mahon CS, Goetz DH, O'Malley JM, Gallagher DM, Zhou M, Murray PG, Craik CS, Tuohy MG Inhibition of a secreted glutamic peptidase prevents growth of the fungus *Talaromyces emersonii*. J Biol Chem. 2008;283(43):29186-95. doi:10.1074/jbc.M802366200. PMID:18687686 18687686 PMC2570871

[23] da Silva RR, Souto TB, de Oliveira TB, de Oliveira LC, Karcher D, Juliano MA, Juliano L, de Oliveira AH, Rodrigues A, Rosa JC, Cabral H Evaluation of the catalytic specificity, biochemical properties, and milk clotting abilities of an aspartic peptidase from *Rhizomucor miehei*. J Ind Microbiol Biotechnol. 2016;43(8):1059-1069. doi:10.1007/s10295-016-1780-4. PMID:27165660 27165660

[24] da Silva RR, de Oliveira LC, Juliano MA, Juliano L, de Oliveira AH, Rosa JC, Cabral H Biochemical and milk–clotting properties and mapping of catalytic subsites of an extracellular aspartic peptidase from basidiomycete fungus *Phanerochaete chrysosporium*. Food Chem. 2017;225:45-54. doi:10.1016/j.foodchem.2017.01.009. PMID:28193432 28193432

[25] Biaggio RT, Silva RR, Rosa NG, Leite RS, Arantes EC, Cabral TP, Juliano MA, Juliano L, Cabral H Purification and biochemical characterization of an extracellular serine peptidase from *Aspergillus terreus*. Prep Biochem Biotechnol. 2016;46(3):298-304. doi:10.1080/10826068.2015.1031387. PMID:25830777 25830777

[26] Hamin Neto YAA, de Oliveira LC, de Oliveira AHC, Rosa JC, Juliano MA, Juliano L, Rodrigues A, Cabral H Determination of specificity and biochemical characteristics of neutral protease isolated from *Myceliophthora thermophila*. Protein Pept Lett. 2015;22(11):972-82. doi:10.2174/0929866522666150817093719. PMID:26279477 26279477

[27] Graminho ER., Da Silva RR, de Freitas Cabral TP, Arantes EC, Da Rosa NG, Juliano L, Okamoto DN, de Oliveira LC, Kondo MY, Juliano MA, Cabral H Purification, characterization, and specificity determination of a new serine protease secreted by *Penicillium waksmanii*. Appl Biochem Biotechnol. 2013;169(1):201-14. doi:10.1007/s12010-012-9974-3. PMID:23179282 23179282

[28] Zanphorlin LM, Cabral H, Arantes E, Assis D, Juliano L, Juliano MA, Da Silva R, Gomes E Purification and characterization of a new alkaline serine protease from the thermophilic fungus *Myceliophthora* sp. Process Biochem. 2011;46(11):2137-2143. doi:10.1016/j.procbio.2011.08.014.

[29] Hamin Neto YAA, de Oliveira LC, de Oliveira JR, Juliano MA, Juliano L, Arantes EC, Cabral H Analysis of the specificity and biochemical characterization of metalloproteases isolated from *Eupenicillium javanicum* using fluorescence resonance energy transfer peptides. Front Microbiol. 2016;7:1-13 PMID:26834723 28119672 10.3389/fmicb.2016.02141 PMC5220088

[30] Merheb–Dini C, Cabral H, Leite RS, Zanphorlin LM, Okamoto DN, Rodriguez GO, Juliano L, Arantes EC, Gomes E, da Silva R Biochemical and functional characterization of a metalloprotease from the thermophilic fungus *Thermoascus aurantiacus*. J Agric Food Chem. 2009;57(19):9210-7. doi:10.1021/jf9017977. PMID:19746980 19746980

[31] da Silva RR, de Oliveira LC, Juliano MA, Juliano L, Rosa JC, Cabral H Activity of a peptidase secreted by *Phanerochaete chrysosporium* depends on lysine to subsite S^'^1. Int J Biol Macromol. 2017;94(Pt A):474-483. doi:10.1016/j.ijbiomac.2016.10.063. PMID:27771408 27771408

[32] Murphy PM, Bolduc JM, Gallaher JL, Stoddard BL, Baker D Alteration of enzyme specificity by computational loop remodeling and design. Proc Natl Acad Sci U S A. 2009;106(23):9215-9220. doi:10.1073/pnas.0811070106. PMID:19470646 19470646 PMC2685249

[33] Adrio JL, Demain A Microbial enzymes: tools for biotechnological processes. Biomolecules. 2014;4(1):117-139. doi:10.3390/biom4010117. PMID:24970208 24970208 PMC4030981

[34] Tavano OL Protein hydrolysis using proteases: an important tool for food biotechnology. J Mol Catal B: Enzym. 2013;90:1-11. doi:10.1016/j.molcatb.2013.01.011.

[35] Park YW, Nam MS Bioactive peptides in milk and dairy products: a review. Korean J Food Sci Anim Resour. 2015;35(6):831-840. doi:10.5851/kosfa.2015.35.6.831. PMID:26877644 26877644 PMC4726964

[36] Guerrero JL, O'Malley MA, Daugherty PS Intracellular FRET–based screen for redesigning the specificity of secreted proteases. ACS Chem Biol. 2016;11(4):961-970. doi:10.1021/acschembio.5b01051. PMID:26730612 26730612 PMC13012646

[37] Giansanti P, Tsiatsiani L, Low TY, Heck AJ Six alternative proteases for mass spectrometry–based proteomics beyond trypsin. Nat Protoc. 2016;11(5):993-1006. doi:10.1038/nprot.2016.057. PMID:27123950 27123950

